# Ectopic Adipose Tissue Depots and Metabolic-Associated Fatty Liver Disease: Relationships With Vitamin D

**DOI:** 10.14740/jocmr6709

**Published:** 2026-10-23

**Authors:** Anna Bragina, Yulia Rodionova, Maria Vetluzhskaya, Maria Vasilchenko, Irakli Loriya, Daria Akvitskaya, Sarvar Abduganiev, Olga Belaya, Karina Umbetova, Andrey Gorobchenko, Danila Voevodin, Valeriy Podzolkov

**Affiliations:** a Department of Faculty Therapy No. 2, I. M. Sechenov First Moscow State Medical University of the Ministry of Health of the Russian Federation (Sechenov University), Moscow, Russia; b Institute of Personalized Cardiology, I. M. Sechenov First Moscow State Medical University of the Ministry of Health of the Russian Federation (Sechenov University), Moscow, Russia; c Department of Infectious Diseases, I. M. Sechenov First Moscow State Medical University of the Ministry of Health of the Russian Federation (Sechenov University), Moscow, Russia

**Keywords:** Metabolic-associated fatty liver disease, Epicardial adipose tissue, Perivascular adipose tissue, Extra-media thickness, Vitamin D

## Abstract

**Background:**

The relationship between vitamin D status, adipose tissue dysfunction and the development of metabolic-associated fatty liver disease (MAFLD) is currently under active investigation. This study aimed to assess the associations of serum vitamin D levels with MAFLD and different ectopic adipose tissue depots, including perivascular adipose tissue (PVAT), epicardial adipose tissue (EAT), and hepatic steatosis, and to determine whether these associations persisted after adjustment for potential confounders.

**Methods:**

This cross-sectional study included 303 patients without established atherosclerotic cardiovascular disease (mean age 55 years (40–60)). PVAT was evaluated by measuring the common carotid artery extra-media thickness (CCA EMT) using duplex ultrasound. EAT thickness was assessed by echocardiography. Hepatic steatosis was quantified by measuring the attenuation coefficient (AC) using vibration-controlled transient elastography. Serum 25-OH vitamin D levels were determined using enzyme-linked immunosorbent assay. Patients were stratified into four groups according to serum vitamin D levels. Multivariable logistic and linear regression analyses were performed to account for potential confounders.

**Results:**

Hepatic steatosis was detected in 125 (41.3%) patients, and MAFLD was detected in 110 (36.3%). Significant inverse correlations were observed between vitamin D levels and AC (r = −0.238) and MAFLD (r = −0.242) (both P < 0.05). General and abdominal obesity were significantly more prevalent in patients with severe vitamin D deficiency compared with those with vitamin D insufficiency and target levels (50% vs. 26.8% and 23%; 70% vs. 29.3% and 23.1%, respectively; P = 0.001 for both). MAFLD was also significantly more frequent in the severe deficiency group than in the deficiency, insufficiency, and target level groups (80% vs. 31.3%, 34.1%, and 15.4%; P = 0.001). In adjusted analyses, the association between 25(OH)D and MAFLD was attenuated after adjustment for body mass index but remained significant in the model including waist circumference. Compared with patients with target vitamin D levels, those with severe deficiency exhibited greater EAT thickness, CCA EMT, and AC (all P < 0.05). Higher 25(OH)D concentrations were independently associated with lower CCA EMT, AC, and EAT thickness after adjustment for age, sex, and adiposity.

**Conclusion:**

Our findings demonstrate associations between vitamin D deficiency, ectopic adipose tissue depots, and MAFLD, suggesting that low vitamin D levels may potentially be linked to adipose tissue dysfunction and cardiometabolic remodeling.

## Introduction

Metabolic-associated fatty liver disease (MAFLD), formerly known as non-alcoholic fatty liver disease (NAFLD), represents a global medical and social challenge, closely linked to the pandemics of obesity and metabolic syndrome. According to systematic reviews and meta-analyses, the prevalence of MAFLD in the general population ranges from 16.1% to 38.8% [1, 2], while among patients with obesity and type 2 diabetes mellitus (T2DM), it exceeds 70% [3]. MAFLD is an independent risk factor for the development of atherosclerosis-associated cardiovascular disease (ASCVD), even after adjustment for traditional risk factors [4].

In recent years, significant attention in the pathogenesis of cardiometabolic disorders in MAFLD is given to perivascular adipose tissue (PVAT), which is involved in the regulation of vascular tone through paracrine mechanisms. In a 2023 consensus statement, European Society of Cardiology emphasized the role of PVAT as a “biosensor” of vascular inflammation and a promising therapeutic target in ASCVD treatment [5]. In obesity, PVAT and other periorgan fat depots (e.g., epicardial adipose tissue (EAT)) acquire a pro-inflammatory phenotype. This phenotype is characterized by increased secretion of pro-inflammatory cytokines such as interleukin-6, interleukin-8, monocyte chemoattractant protein-1, etc., oxidative stress, and reduced synthesis of the anti-inflammatory adipokine, adiponectin [6]. Alongside the development of meta-inflammation, there is a reduction in nitric oxide synthesis, increased expression of extracellular matrix components, and proliferation of vascular smooth muscle cells. These processes lead to increased arterial stiffness and the development of atherosclerosis and ASCVD [7]. Furthermore, there is a reduction in adipocyte storage capacity, resulting in the redirection of lipid flow to other organs and tissues, including the ectopic accumulation of fat in the liver [8].

However, ectopic fat accumulation alone does not fully explain the development of MAFLD. Its pathogenesis is multifactorial and involves complex interactions between adipose tissue dysfunction, insulin resistance, inflammatory and metabolic disturbances, genetic susceptibility, dietary and lifestyle factors, and micronutrient status [8, 9]. Among potentially relevant nutritional factors, current research actively investigates vitamin D as a potential driver of adipose tissue dysfunction and MAFLD [9]. Vitamin D deficiency (serum 25(OH)D < 20 ng/mL) is detected in 29–54% of patients with MAFLD and correlates with the degree of hepatic steatosis and fibrosis [10]. Importantly, restoring normal vitamin D levels has been shown to decrease the production of pro-inflammatory cytokines in visceral fat [11].

Despite growing attention to the potential role of vitamin D in obesity, adipose tissue dysfunction, and MAFLD [9–11], the relationship between vitamin D status and different ectopic adipose tissue depots remains insufficiently characterized. Limited data are available on the simultaneous associations of serum 25(OH)D with different ectopic fat depos, including MAFLD, at the same time within the same clinical cohort. Furthermore, it remains unclear to what extent these relationships are independent of major demographic characteristics and general or abdominal adiposity. This issue is particularly relevant in individuals without established ASCVD, in whom alterations in ectopic adipose tissue may occur before clinically manifest cardiovascular disease.

Therefore, the aim of this study was to assess the associations of vitamin D levels with MAFLD and different ectopic adipose tissue depots, including PVAT assessed by common carotid artery extra-media thickness (CCA EMT), EAT, and hepatic steatosis assessed by ultrasound attenuation coefficient (AC), in individuals without established ASCVD, and to determine whether these associations persisted after adjustment for potential relevant confounders.

## Materials and Methods

### Study design and participants

This cross-sectional study included 303 patients without ASCVD (152 men and 151 women; mean age 55 (40–60) years), who were admitted to the Therapy Department of University Clinical Hospital No. 4, affiliated with Sechenov University. The study was performed in compliance with the principles of the Declaration of Helsinki. Approval was granted by the Local Ethics Committee (Protocol No. 28-24, dated November 21, 2024).

Eligible participants were aged 30 to 80 years and provided written informed consent for study participation.

Exclusion criteria were: excessive alcohol consumption (> 30 g/day for men and > 20 g/day for women) [12] or alcohol use disorder (AUDIT score > 8), regular statin use over the past year, serological evidence of viral hepatitis or other chronic liver diseases, symptomatic hypertension (HTN), clinical manifestations of atherosclerosis (including coronary artery disease, cerebrovascular disease), estimated glomerular filtration rate (eGFR) < 60 mL/min/1.73 m^2^, proteinuria ≥ 300 mg/day, type 1 diabetes mellitus and T2DM, systemic autoimmune diseases, inflammatory diseases of any localization, oncology, pregnancy, and psychiatric disorders.

### Clinical and anthropometric assessment

All patients underwent assessment of anthropometric parameters (height, weight, waist circumference (WC)), and body mass index (BMI). Overweight, general obesity, and its grade were determined in accordance with the multidisciplinary national clinical guidelines “Treatment of Obesity and Comorbid Diseases,” 2021 [13]. WC ≥ 102 cm for men and ≥ 88 cm for women was used as the criterion for abdominal obesity (AO) [14]. Dyslipidemia was diagnosed based on the “Lipid Metabolism Disorders” national guidelines, 2023 [15]. HTN and its staging were assessed following the 2024 Russian Society of Cardiology recommendations [16].

### Assessment of PVAT

PVAT was assessed using the CCA EMT according to the method described by Skilton et al [17]. Duplex ultrasound examination of the brachiocephalic arteries was performed using an L12-3 linear transducer on a PHILIPS EPIQ 7 ultrasound system. CCA EMT was measured in the longitudinal plane 1.5 cm proximal to the carotid bifurcation as the distance between the inner surface of the posterior wall of the internal jugular vein and the outer surface of the “intima-media” complex of the anterior CCA wall [17]. Measurements were obtained bilaterally, and the mean value of the right and left CCA EMT measurements was used for subsequent analysis. According to data obtained in our previous study, a CCA EMT value ≥ 0.54 mm was considered indicative of increased PVAT accumulation [18].

### Assessment of EAT

EAT thickness was assessed by transthoracic echocardiography using an X5-1 sector transducer. EAT was measured over the free wall of the right ventricle in the parasternal view as the echo-free space between the outer myocardial wall and the visceral layer of the pericardium. Measurements were performed at end-systole over 10 consecutive cardiac cycles.

Age-specific cut-off values proposed by Kuznetsova et al were used to identify increased EAT thickness: ≥ 5 mm in participants aged < 45 years, ≥ 6 mm in those aged 45–55 years, and ≥ 7 mm in participants aged > 55 years [19].

### Assessment of hepatic steatosis

Hepatic steatosis was identified and quantitatively assessed by measuring the AC. Measurements were performed using a PHILIPS EPIQ 7 ultrasound system (USA, 2018) equipped with the “Liver Fat Quantification” software package, a C5-1 convex transducer, and the “Elasto” software module in the morning after overnight fasting, with the participant in the supine position and the right arm placed behind the head. Data were obtained during breath-holding after a shallow expiration. The region of interest was positioned in segments VII–VIII of the right hepatic lobe approximately 2 cm below the liver capsule, avoiding large vessels, focal lesions, and other structures that could interfere with the measurement. All examinations were performed in accordance with the World Federation for Ultrasound in Medicine and Biology guidelines on Liver Multiparametric Ultrasound [20]. An AC > 0.67 dB/cm/MHz was considered indicative of steatosis [21].

### Definition of MAFLD

MAFLD was defined according to the international expert consensus criteria proposed by Eslam et al [22]. The diagnosis required evidence of hepatic steatosis (in the absence of excessive alcohol consumption (> 30 g/day for men and > 20 g/day for women) or other acute and chronic liver diseases) together with at least one of the following: overweight/obesity, T2DM, or metabolic dysregulation.

Because patients with T2DM were excluded from the present study, participants with hepatic steatosis were classified as having MAFLD if they had overweight/obesity or, in participants without overweight/obesity, if metabolic dysregulation was present according to the consensus criteria: AO, HTN/antihypertensive treatment, dyslipidemia, impaired glucose metabolism, or elevated high-sensitivity C-reactive protein levels. Metabolic dysregulation was defined by the presence of at least two metabolic risk abnormalities according to the Eslam et al criteria [22].

### Assessment of vitamin D status

Serum vitamin D levels were determined in all patients using the enzyme-linked immunosorbent assay method with the “DRG 25-OH Vitamin D ELISA” kit (DRG Instruments GmbH, Germany). Blood sampling and assessment of serum vitamin D levels were performed during the autumn season for all participants. None of the included patients received vitamin D supplementation at the time of the study. Patients were divided into groups based on their vitamin D status according to the national clinical guidelines of the Russian Endocrine Society, considering the geographic and climatic characteristics (reduced sunlight exposure during a substantial part of the year) of the Russian population [23].

### Statistical analysis

Statistical analysis was performed using Statistica 12.0 (StatSoft Inc., USA). The distribution of continuous variables was assessed using the Shapiro–Wilk test. Normally distributed variables are presented as mean ± standard deviation (SD), non-normally distributed variables are presented as median and interquartile range (Q1–Q3). Categorical variables are presented as absolute numbers and percentages. Correlations were analyzed using Spearman’s rank correlation coefficient. Categorical variables were compared using Pearson’s chi-square test. Continuous variables were compared between two independent groups using the Mann–Whitney U test and among multiple independent groups using the Kruskal–Wallis test. When an overall comparison among multiple groups was statistically significant, *post-hoc* pairwise comparisons were performed with adjustment for multiple testing. Multivariable binary logistic regression was used for analyses with a binary dependent variable. Multiple linear regression analysis was used for continuous dependent variables. Models were adjusted for potential confounders. All statistical tests were two-sided, and a P value <0.05 was considered statistically significant.

## Results

Clinical and demographic characteristics of the examined patients are presented in Table 1.

**Table 1:** Clinical and Demographic Characteristics of the Examined Patients

Parameter	Value
Age, years (Me (Q1–Q3))	55 (40–60)
Sex male/female, n (%)	152/151 (50.2/49.8)
BMI, kg/m^2^	27.8 (24.7–32.7)
WC	
Men, cm (Me (Q1–Q3))	100 (92–110)
Women, cm (Me (Q1–Q3))	94 (80–105)
Cardiovascular risk factors	
Hypertension, n (%)	151 (49.8)
Overweight (25 ≤ BMI < 30 kg/m^2^), n (%)	100 (33.0)
Obesity (BMI ≥ 30 kg/m^2^), n (%)	118 (38.9)
Obesity grade 1/2/3, %	58.5/26.3/15.2
Abdominal obesity, n (%)	153 (50.5)
Dyslipidemia, n (%)	141 (46.5)
Hypercholesterolemia, n (%)	114 (37,6)
Hypertriglyceridemia, n (%)	106 (35)
Smoking, yes/no, n (%)	106 (34.9)
Ectopic adipose tissue	
EAT thickness, mm (Me (Q1–Q3))	6.7 (4.9–8.4)
CCA EMT, mm (Me (Q1–Q3))	0.6 (0.5–0.7)
Attenuation coefficient, dB/cm/MHz (Me (Q1–Q3))	0.64 (0.59–0.72)
Hepatic steatosis, n (%)	125 (41.3)
MAFLD, n (%)	110 (36.3)

BMI: body mass index; CCA EMT: common carotid artery extra-media thickness; EAT: epicardial adipose tissue; MAFLD: metabolic-associated fatty liver disease; Me: median; WC: waist circumference.

The examined cohort was equally represented by men (50.2%) and women (49.8%). In the overall study population, the prevalence of HTN, smoking, dyslipidemia, and AO corresponded to general population data [24–26]. Hepatic steatosis was detected in 125 (41.3%) patients. Of these, 110 (36.3%) patients met the diagnostic criteria for MAFLD. Among patients with MAFLD, the diagnosis was established based on the presence of hepatic steatosis combined with overweight in 34 patients and with obesity in 76 patients. Among these patients, HTN was present in all cases, while dyslipidemia was observed in 87 patients, including hypercholesterolemia in 28 and hypertriglyceridemia in 60 patients.

Analysis of the association between anthropometric parameters, vitamin D levels, and indicators of ectopic adipose tissue depots revealed statistically significant correlations MAFLD with WC (r = 0.688, P < 0.05), CCA EMT (r = 0.645, P < 0.05), and EAT (r = 0.53, P < 0.05). A statistically significant inverse correlations were found between vitamin D level and the AC (r = −0.238, P < 0.05), and the presence of MAFLD (r = −0.242, P < 0.05), indicating a link between vitamin D status and dysfunctional adipose tissue remodeling (Fig. 1).

**Figure 1:**
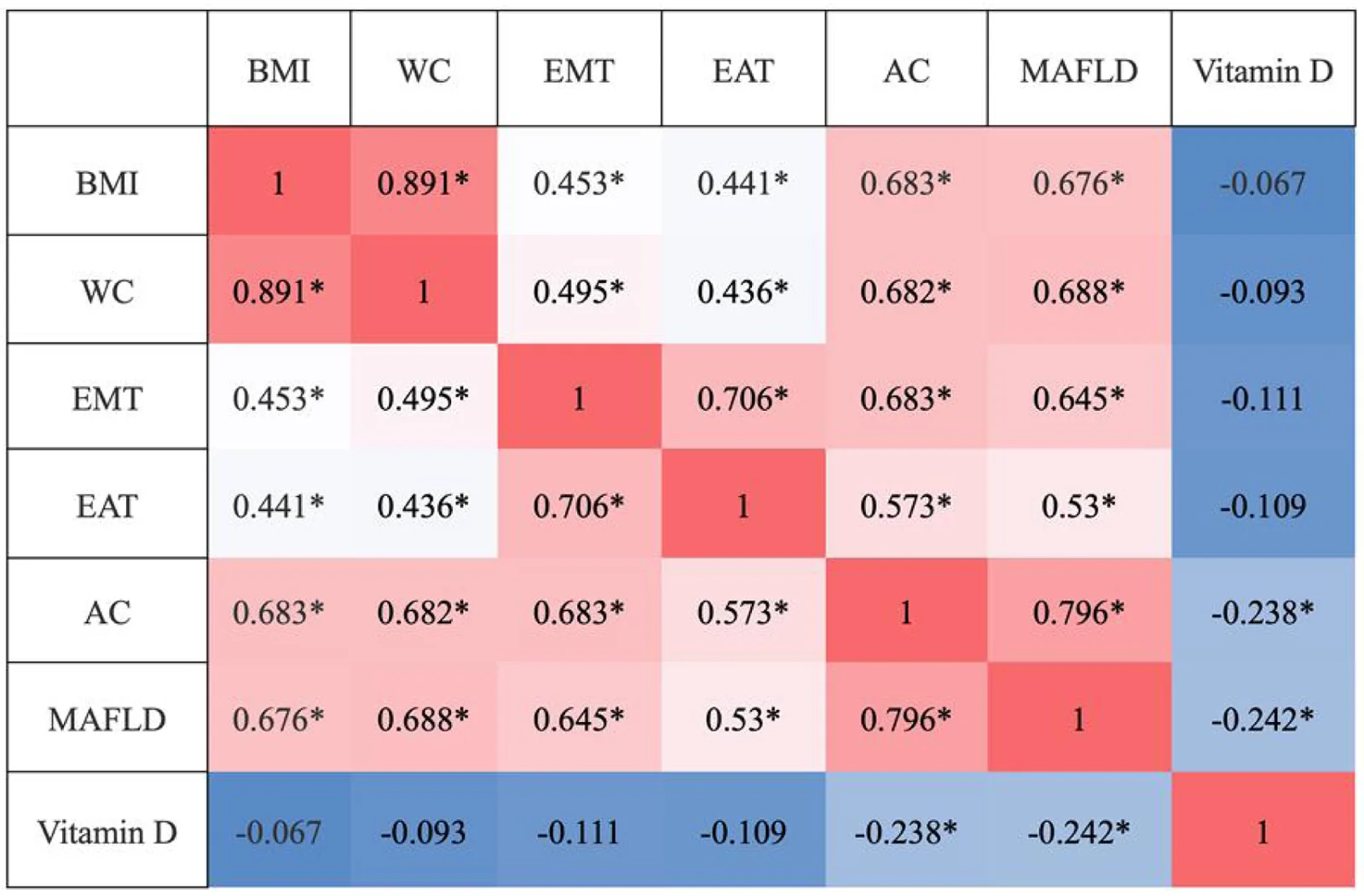
Correlations between anthropometric parameters, vitamin D levels, and ectopic adipose tissue measures. AC: attenuation coefficient; BMI: body mass index; EAT: epicardial adipose tissue; EMT: extra-media thickness; MAFLD: metabolic-associated fatty liver disease; WC: waist circumference.

All examined patients were divided into groups based on their serum vitamin D (25(OH)D) level: severe deficiency (< 10 ng/mL) (n = 38); deficiency (10–19 ng/mL) (n = 61); insufficiency (20–29 ng/mL) (n = 155); and optimal level (30–60 ng/mL) (n = 49) (Fig. 2) [23].

**Figure 2:**
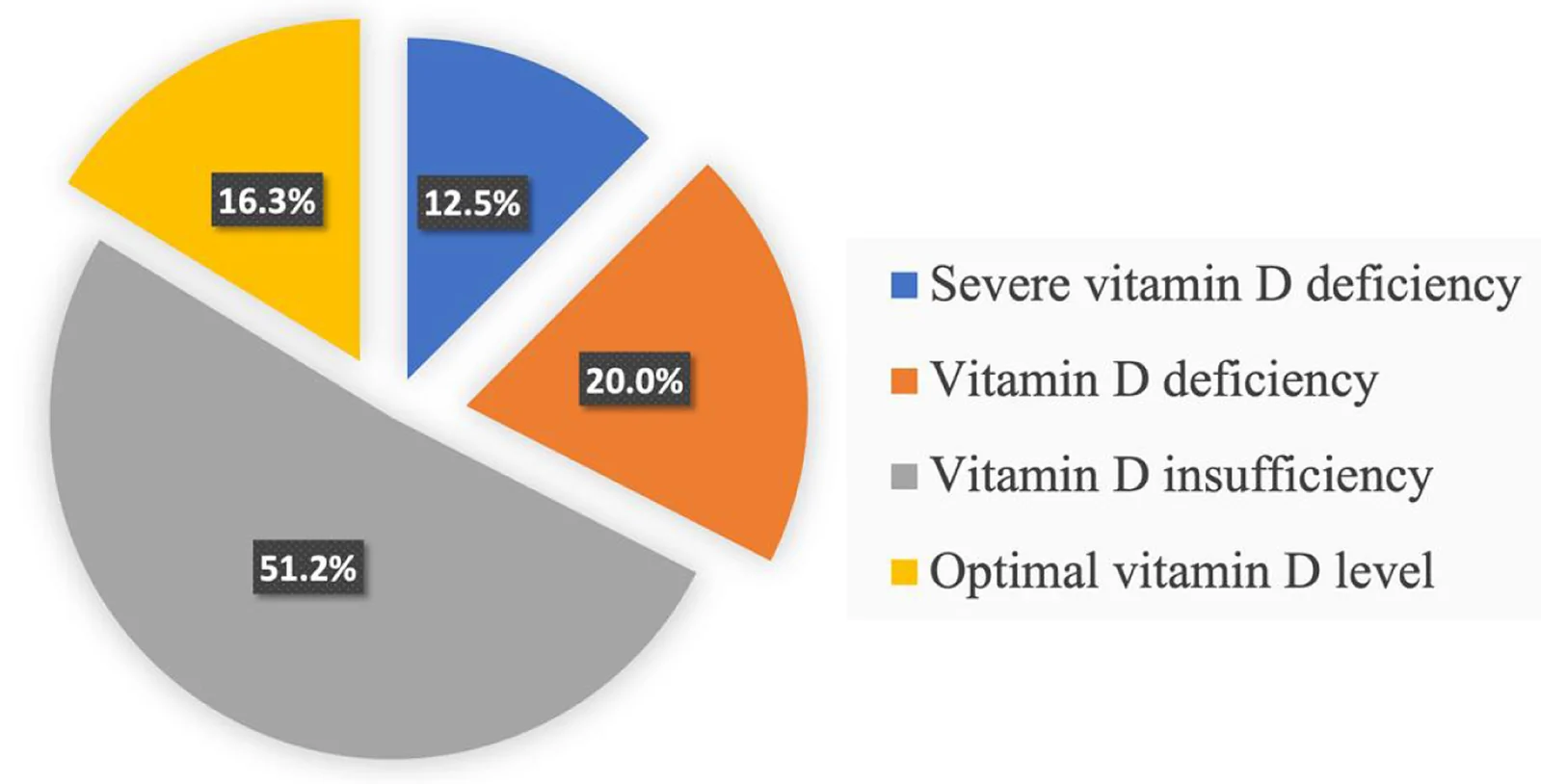
Distribution of patients according to vitamin D levels.

We analyzed the prevalence of general obesity (by BMI) and AO (by WC) according to vitamin D levels. Both conditions were statistically significantly more frequent in patients with severe vitamin D deficiency compared to those with vitamin D insufficiency and optimal vitamin D levels: 50% vs. 26.8% and 23% (P = 0.001) for general obesity, and 70% vs. 29.3% and 23.1% (P = 0.001) for AO, respectively (Fig. 3).

**Figure 3:**
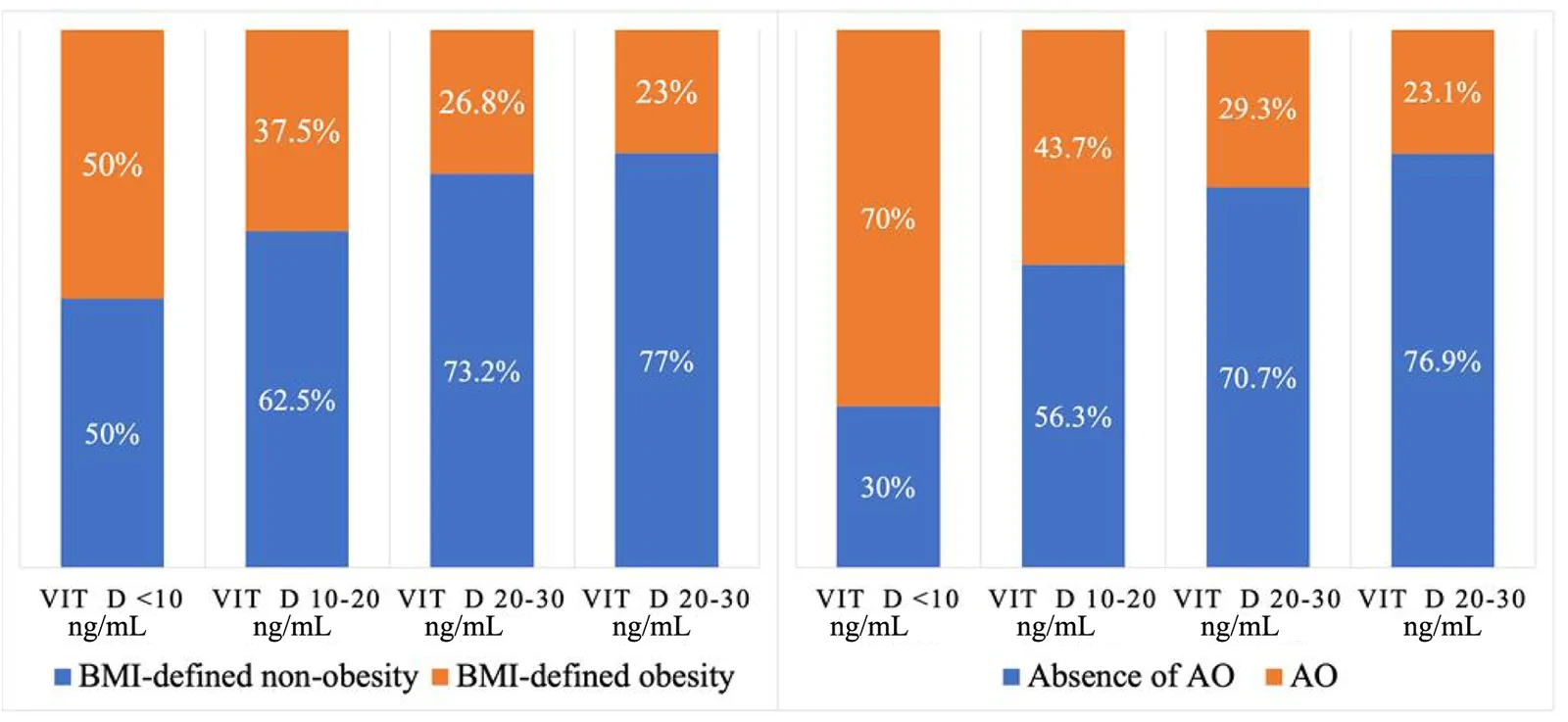
Prevalence of BMI-defined obesity and AO depending on vitamin D status in the examined patients. AO: abdominal obesity; BMI: body mass index.

A statistically significantly higher frequency of MAFLD was found in patients with severe vitamin D deficiency compared to the groups with vitamin D deficiency, insufficiency, and optimal levels: 80% vs. 31.3%, 34.1%, and 15.4% (P = 0.001), respectively (Fig. 4).

**Figure 4:**
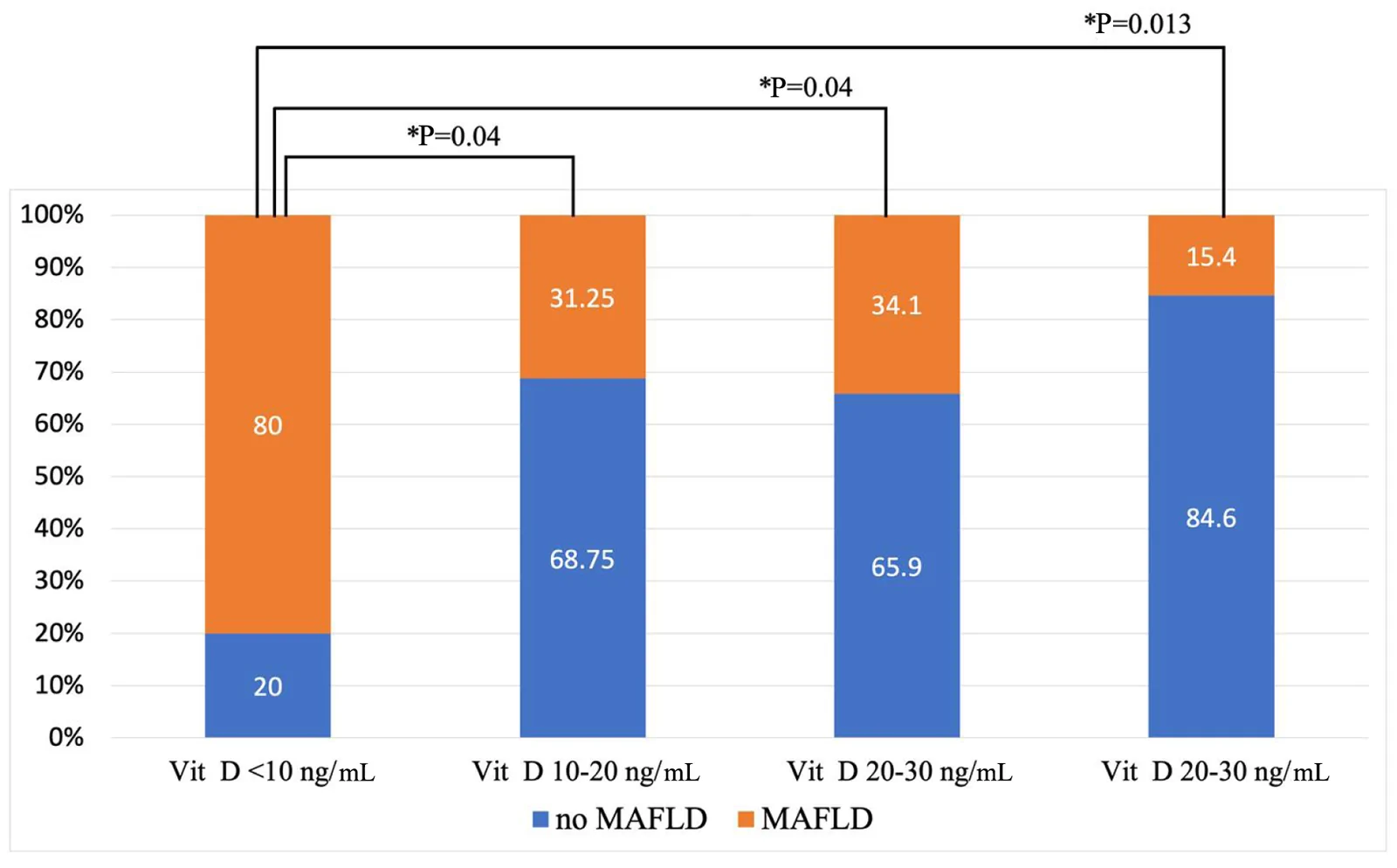
Prevalence of MAFLD depending on vitamin D status in the examined patients. MAFLD: metabolic-associated fatty liver disease.

To determine whether the association between vitamin D status and MAFLD remained independent of potential confounding factors, multivariable logistic regression analyses were performed (Table 2). Because BMI and WC were strongly correlated (r = 0.891), they were included in separate models to avoid multicollinearity. Physical activity and medication use were not included in the final models because these variables showed no variability among the study participants. Patients with T2DM were not included in the study cohort. HTN was excluded from the final models due to unstable estimates caused by complete separation, as all patients with MAFLD had concomitant hypertension.

**Table 2:** Multivariable Logistic Regression Analysis of Factors Associated With MAFLD, Including Vitamin D Status, BMI, and WC

Variables	β coefficient	OR	95% CI	P value
Model 1				
Constant	−8.545	-	-	0.002
Age	0.105	1.111	1.044–1.182	< 0.001
Sex	−1.507	0.221	0.028–1.732	0.151
Dyslipidemia	1.035	2.817	0.539–14.714	0.220
Smoking	0.019	1.019	0.157–6.610	0.984
BMI	0.182	1.20	1.065–1.352	0.003
Vitamin D	−0.110	0.896	0.802–1.000	0.05
Model 2				
Constant	−8.545	-	-	0.005
Age	0.204	1.227	1.051–1.431	< 0.009
Sex	−1.385	0.250	0.005–11.112	0.474
Dyslipidemia	3.073	11.602	0.892–13.055	0.059
Smoking	−2.095	0.123	0.002–5.316	0.276
WC	0.429	1.536	1.144–2.061	0.004
Vitamin D	−0.211	0.810	0.662–0.990	0.039

BMI: body mass index; CI: confidence interval; MAFLD: metabolic-associated fatty liver disease; Me: median; OR: odds ratio; WC: waist circumference.

In the model adjusted for age, sex, smoking status, dyslipidemia, and BMI, vitamin D was no longer significantly associated with MAFLD (odds ratio (OR) 0.90, 95% confidence interval (CI) 0.80–1.00, P = 0.050), while BMI remained an independent predictor of MAFLD (OR 1.20, 95% CI 1.07–1.35, P = 0.003). However, when BMI was replaced by WC, vitamin D remained independently associated with MAFLD (OR 0.81, 95% CI 0.66–0.99, P = 0.039), together with WC (OR 1.54, 95% CI 1.14–2.06, P = 0.004) and age (OR 1.23, 95% CI 1.05–1.43, P = 0.009).

Analysis of ectopic adipose tissue depots (EAT, CCA EMT, AC) across vitamin D levels showed that they were significantly bigger in the severe vitamin D deficiency group compared to the optimal-level group: EAT (8 (7.1–10.1) vs. 5.8 (4.6–9.5), P = 0.03); CCA EMT (0.7 (0.6–0.9) vs. 0.6 (0.5–0.7), P = 0.013); AC (0.72 (0.67–0.76) vs. 0.61 (0.58–0.71), P = 0.003) (Fig. 5). In multivariable linear regression analyses, higher serum 25(OH)D concentrations remained independently associated with lower CCA EMT, AC, and EAT thickness after adjustment for age, sex, and adiposity. The associations were consistent in alternative models including either BMI or WC and were modest in magnitude (standardized β ranging from −0.100 to −0.142; all P < 0.05). No substantial multicollinearity was observed (VIF ≤ 1.23).

**Figure 5:**
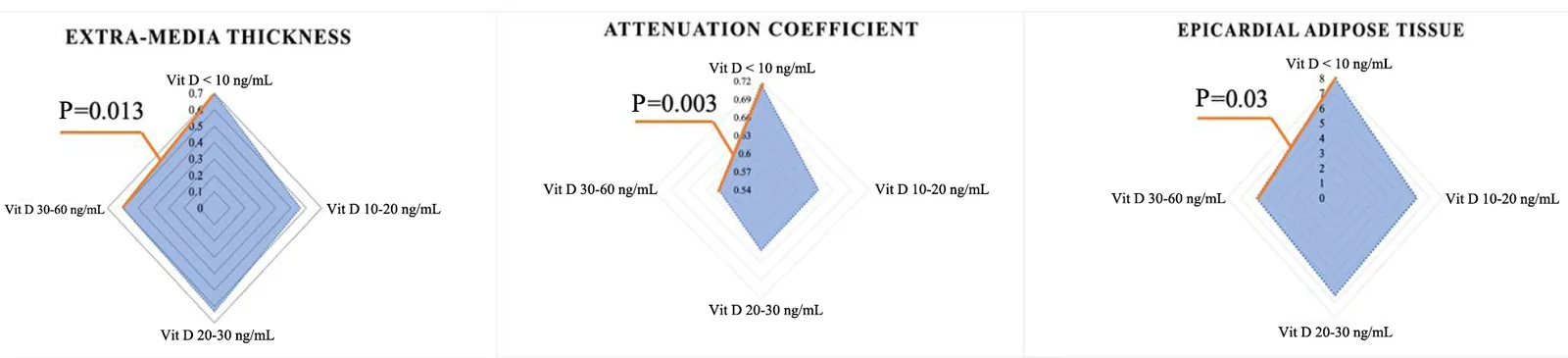
Ectopic fat depots depending on vitamin D status in the examined patients.

## Discussion

To investigate the associations between vitamin D status and ectopic adipose tissue depots, we assessed the relationships between this micronutrient and markers of adipose tissue dysfunction.

In our study, serum vitamin D levels demonstrated statistically significant inverse correlations with AC values and the presence of MAFLD, while ectopic adipose tissue measures, including common CCA EMT and EAT thickness, were positively correlated with anthropometric and metabolic parameters associated with obesity and hepatic steatosis. These findings suggest close associations between vitamin D status, ectopic adipose tissue depots, and cardiometabolic abnormalities.

We found that the prevalence of general obesity (by BMI) and AO was statistically significantly higher in individuals with severe vitamin D deficiency compared to patients with insufficiency or target vitamin D levels (50% vs. 26.8% and 23%, P = 0.001; 70% vs 29.3% and 23.1%, P = 0.001, respectively). Similar results were reported in several meta-analyses [27, 28], which discussed the link between vitamin D and obesity. For example, in a meta-analysis by Hajhashemy et al (2021) including 36 studies (n = 257,699), the highest serum vitamin D level was associated with 23% reduced odds of AO compared to the lowest level of this micronutrient (OR 0.77; 95% CI 0.71–0.83) [27]. Furthermore, a dose–response analysis showed that each 25 nmol/L increase in serum vitamin D was associated with an 8% reduction in the risk of AO (OR 0.92; 95% CI 0.85–0.99). In another meta-analysis by Rafiq et al (2018), which analyzed 55 studies, an inverse association was found between serum vitamin D levels and BMI both in patients with diabetes (r = −0.173, 95% CI −0.241 to −0.103, P = 0.001) and in patients without diabetes (r = −0.152, 95% CI −0.187 to −0.116, P = 0.001) [28]. It is important to note that this large systematic review and meta-analysis included studies involving patients with diabetes, and studies conducted on the Russian population were not included. Interesting results were obtained in a study by Walsh et al (2016) (n = 223), which found an inverse correlation between vitamin D levels and BMI only in autumn, spring, and winter. Additionally, statistically significantly lower vitamin D concentrations were observed in individuals with obesity and overweight compared to those with normal weight (45.0 and 40.8 nmol/L vs. 58.6 nmol/L, P < 0.001) [29].

Attention should be paid to MAFLD as a component of metabolic syndrome and a central link in cardiometabolic relationships. Our study demonstrated that patients with severe vitamin D deficiency had a statistically significantly higher prevalence of MAFLD compared to groups with vitamin D deficiency, insufficiency, and optimal levels: 80% vs. 31.3%, 34.1%, and 15.4% (P = 0.001). The differential findings observed after adjustment for BMI and WC may reflect the distinct aspects of adiposity represented by these parameters. While BMI reflects overall adiposity, WC is a marker of central fat accumulation and abdominal obesity. The persistence of the association between lower vitamin D levels and MAFLD after adjustment for WC suggests that this relationship may not be fully explained by abdominal obesity alone. These findings support the hypothesis that vitamin D deficiency may be associated with MAFLD through additional, partly adiposity-independent hepatic pathways. Potential mechanisms include impaired fibroblast growth factor-21 signaling, activation of hepatic stellate cells, enhanced transforming growth factor-β (TGF-β)/SMAD-mediated profibrotic signaling, and progression of hepatic fibrogenesis. Experimental data indicate that vitamin D receptor signaling may modulate hepatic stellate cell activation and counteract TGF-β-driven fibrogenic responses, supporting a potential mechanistic link between vitamin D deficiency and adverse liver remodeling [30, 31]. Therefore, low vitamin D levels may represent not only a marker of an unfavorable metabolic profile but also a factor linked to partially independent mechanisms involved in MAFLD development. In this context, vitamin D status may be considered a potential therapeutic target, with further prospective and interventional studies needed to confirm this assumption.

Supporting these findings, a large population-based study of 12,878 participants found an association between vitamin D insufficiency and increased odds of MAFLD (OR 1.130; 95% CI 1.035–1.234) [32]. The authors noted that the mean serum vitamin D level progressively decreased with an increasing number of metabolic risk factors. They emphasize the potential role of vitamin D in systemic inflammation, regulation of hepatic lipid metabolism, maintenance of insulin sensitivity, and inhibition of fibrosis through suppression of the TGF-β signaling pathway in hepatic stellate cells.

Scientific statements from the American Heart Association [33] and consensus documents from the European Society of Cardiology [5] highlight the diagnostic significance of periorgan adipose tissue depots in cardiovascular remodeling, attributing this to their high humoral activity. The relationship between ectopic adipose tissue dysfunction and vitamin D status may potentially be bidirectional. Dysfunctional ectopic adipose tissue may contribute to altered vitamin D metabolism through chronic low-grade inflammation and activation of pro-inflammatory pathways. Supporting this concept, Dozio et al (2015) demonstrated increased inflammatory activity in EAT in patients with coronary artery disease, potentially related to both systemic and local vitamin D deficiency [34]. At the same time, vitamin D deficiency itself may also be associated with adipose tissue dysfunction and ectopic fat remodeling, suggesting a close interaction between these processes. These patterns were observed in our study for ectopic fat depots. Along with a higher AC in severe vitamin D deficiency, EAT and CCA EMT were also statistically significantly higher in this patient group compared to the group with target vitamin D levels. Comparable data were obtained in a study on a Chinese population, where the authors demonstrated an inverse correlation between EAT and vitamin D levels in non-obese patients with T2DM (r = −0.25, P = 0.039) [35]. This indicates an association between periorgan fat accumulation and vitamin D even in the absence of general obesity. It is important to note that studies investigating the association between vitamin D levels and ectopic fat depots (PVAT and EAT) have not been conducted on the Russian population or in cohorts with T2DM.

Thus, the observed associations between vitamin D deficiency, obesity, ectopic adipose tissue depots, and MAFLD may reflect closely interconnected metabolic and inflammatory processes. Lower vitamin D levels are associated with insulin resistance, oxidative stress, increased lipolysis and free fatty acid release, reduced adiponectin secretion, and enhanced production of pro-inflammatory cytokines. Obesity and ectopic adipose tissue depots, including PVAT and EAT, may also contribute to altered vitamin D metabolism through redistribution and sequestration of vitamin D within adipose tissue, as well as increased leptin synthesis. In turn, MAFLD progression may be associated with impaired fibroblast growth factor-21 signaling, activation of hepatic stellate cells, increased TGF-β activity, and hepatic fibrosis. Together, these mechanisms may contribute to the close associations observed between vitamin D status, ectopic adipose tissue remodeling, and MAFLD (Fig. 6).

**Figure 6:**
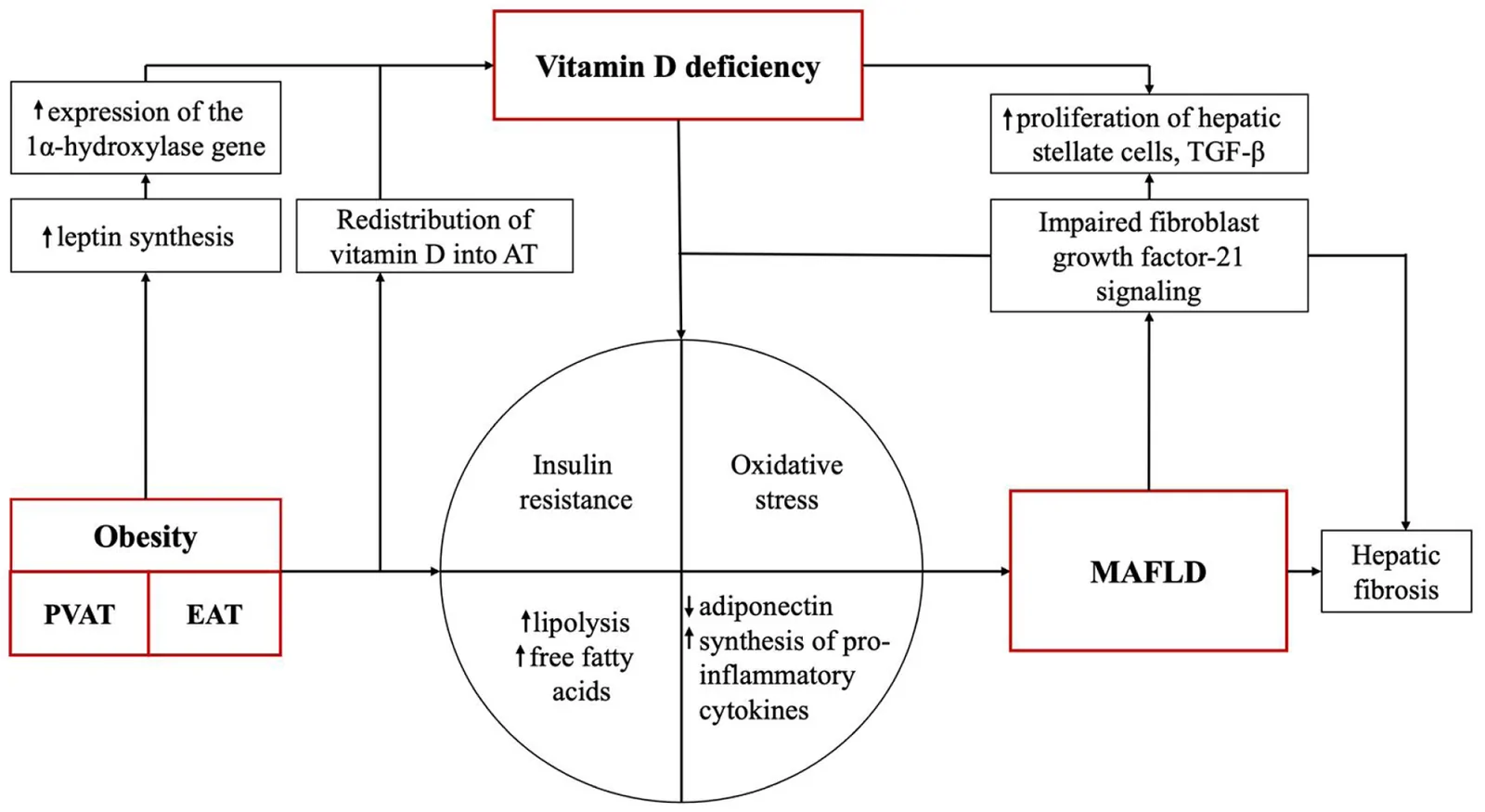
The role of ectopic fat depots and vitamin D deficiency in the development of MAFLD. AT: adipose tissue; EAT: epicardial adipose tissue; MAFLD: metabolic-associated fatty liver disease; PVAT: perivascular adipose tissue.

Several limitations of the present study should be acknowledged. First, the cross-sectional design and relatively small sample size limit the ability to establish causal relationships between vitamin D status, ectopic adipose tissue, and MAFLD. Hepatic steatosis was assessed using the ultrasound AC, which is a practical non-invasive method for estimating hepatic fat content but may be influenced by technical and methodological factors and does not provide the same quantitative information as MRI-based techniques or histological assessment. Similarly, EAT was evaluated by echocardiographic thickness, which is widely used in clinical studies but reflects a linear measure and may not fully characterize the total volume and regional distribution of EAT. The potential contribution of long-standing lipid abnormalities should also be considered. Although dyslipidemia was considered in the multivariable analysis, detailed information on the duration and cumulative severity of hypercholesterolemia and hypertriglyceridemia was not available. Therefore, their possible contribution to hepatic lipid accumulation and the observed metabolic associations cannot be completely excluded. In addition, the association between vitamin D levels and MAFLD was modest and became weaker after adjustment for BMI, suggesting that general adiposity may explain part of this relationship. Finally, lifestyle characteristics were not systematically assessed, and the single-center design and assessment of vitamin D during the autumn season may somewhat limit the generalizability of the results. Nevertheless, the obtained results provide additional data regarding the relationship between vitamin D status, ectopic adipose tissue, and cardiometabolic disorders in the Russian population. Further prospective studies with larger cohorts are needed to better clarify the clinical and pathophysiological significance of these associations.

### Conclusion

Thus, vitamin D should be viewed not only as a biochemical marker responsible for bone remodeling but also vitamin D status may be considered an important component of metabolic and inflammatory processes associated with ectopic adipose tissue remodeling and MAFLD. Further studies are needed to better clarify the relationships between vitamin D deficiency, ectopic adipose tissue depots, and cardiometabolic disorders, as well as their potential clinical significance in cardiovascular risk assessment and management.

## Data Availability

The authors declare that data supporting the findings of this study are available within the article. Any raw data are available from the corresponding author upon reasonable request.

## Abbreviations

AC: attenuation coefficient
AO: abdominal obesity
ASCVD: atherosclerosis-associated cardiovascular disease
BMI: body mass index
CCA: common carotid artery
EAT: epicardial adipose tissue
eGFR: estimated glomerular filtration rate
EMT: extra-media thickness
HTN: hypertension
MAFLD: metabolic-associated fatty liver disease
NAFLD: non-alcoholic fatty liver disease
PVAT: perivascular adipose tissue
T2DM: type 2 diabetes mellitus
WC: waist circumference
